# Functional characterization of *EjAP1-like1* reveals its role in floral development and flowering time regulation in loquat (*Eriobotrya japonica*)

**DOI:** 10.3389/fpls.2025.1713266

**Published:** 2025-12-12

**Authors:** Yifang Wang, Chongbin Zhao, Xiaoyan Yang, Bin Wang, Ze Peng, Xianghui Yang, Chongjian Ma, Yuan Yuan, Yuanyuan Jiang

**Affiliations:** 1 Guangdong Provincial Key Laboratory of Utilization and Conservation of Food and Medicinal Resources in Northern Region/College of Biology and Agriculture, Shaoguan University, Shaoguan, Guangdong, China; 2 College of Horticulture, South China Agricultural University, Guangzhou, Guangdong, China; 3 Guangdong Provincial Engineering and Technology Research Center of Special Fruit and Vegetables in Northern Region, Shaoguan University, Shaoguan, Guangdong, China; 4 State Key Laboratory of Woody Oil Resources Utilization, Central South University of Forestry and Technology, Changsha, China

**Keywords:** loquat, gene cloning, flowering, molecular mechanisms, AP1

## Abstract

APETALA1 (AP1), a member of the MADS-box transcription factor family, plays a pivotal role in floral meristem identity and organ formation. In this study, we identified and cloned a novel AP1 homolog gene, *EjAP1-like*1, from loquat. The gene encodes a 262-amino acid protein with a typical MADS-box domain and shares close phylogenetic relationships with known flowering-related AP1 homologs, suggesting a conserved role in flowering regulation. Promoter analysis revealed multiple cis-acting elements responsive to light, GA, MeJA, and drought. Expression profiling showed that *EjAP1-like1* is highly expressed in the shoot apical meristem during floral induction, and its expression is modulated by GA_3_ treatment and short-day photoperiod. Subcellular localization confirmed its nuclear localization. Moreover, ectopic expression of *EjAP1-like1* in *Arabidopsis thaliana* led to significantly earlier flowering. Collectively, these findings highlight EjAP1-like1 as a potential floral inducer in loquat and offer a useful genetic resource for understanding and manipulating flowering time in woody fruit crops.

## 1 Introduction

Floral bud differentiation is a key biological process marking the transition from vegetative to reproductive growth in angiosperms. The timing of floral induction is not only essential for successful reproduction but also directly influences fruit yield and quality in horticultural crops. The regulatory mechanisms underlying floral induction involve multiple genetic pathways, including the photoperiod, gibberellin (GA), vernalization, thermosensory, autonomous, and age-related pathways (Blümel et al., 2015; Maple et al., 2024). These pathways together form a complex and finely tuned gene regulatory network, in which the MADS-box transcription factor family plays a central role (Becker and Theißen, 2003; De Bodt et al., 2003; Parenicová et al., 2003).

APETALA1 (AP1) is an A-class MADS-box gene originally identified in *Arabidopsis thaliana*. It is expressed in early floral primordia and later becomes confined to sepals and petals, where it determines floral meristem identity and organ patterning (Alejandra Mandel et al., 1992). The AP1 protein forms both homodimers and heterodimers with other MADS-box proteins to regulate the expression of key downstream floral organ identity genes, mediated through its conserved K-domain (Mandel and Yanofsky, 1995; Riechmann et al., 1996; Meng et al., 2025). Recent studies have demonstrated the conserved and diversified roles of AP1 homologs across plant species. In monocot species such as rice and maize, AP1-like genes (e.g., OsMADS14, ZAP1) regulate inflorescence meristem identity and spikelet formation (Mena et al., 1995; Cho et al., 1999; Moon et al., 1999). In woody or herbaceous dicot species such as apple, bamboo, and *Cajanus cajan*, AP1 homologs are involved in floral organ identity and flowering time regulation (Kotoda et al., 2002; Basak et al., 2025; Bhattacharjee et al., 2025).

Loquat (*Eriobotrya japonica*), an evergreen fruit tree of the Rosaceae family, originated in China and has been cultivated for more than 2,000 years. It is now grown in over 30 countries worldwide (Jiang et al., 2022). In addition to its value as a fresh fruit, its fruits, leaves, and flowers possess important medicinal properties, including antitussive, antiasthmatic, antidiabetic, and anti-inflammatory activities, and are widely used in both traditional and modern medicine (Lin et al., 1998; Yang and Peng, 2022). In apple and pear, floral induction occurs in one year and flowering takes place the following spring. In loquat, however, both processes occur within a single year. Its floral initiation typically occurs from late June to early July, while anthesis occurs mainly from October to January (Kurokura et al., 2013; Jiang et al., 2019c; Peng et al., 2022; Jiang et al., 2025). This distinctive flowering behavior, involving floral initiation in autumn and fruit maturation in early spring, reflects the unique physiological features of loquat and reinforces the need to investigate its potentially divergent regulatory mechanism controlling floral development and timing. Previous studies have shown that GA_3_ and IAA concentrations are significantly lower in floral buds than in vegetative buds, and exogenous GA_3_ application suppresses floral induction, promoting vegetative growth (Liu et al., 2007; Jiang et al., 2019a, 2019b, 2019c, 2025). However, the molecular basis of floral induction in loquat remains largely unknown. To date, only one AP1 homolog (EjAP1) has been identified in the cultivar ‘Zaohong No.6’ and was predicted to participate in flower bud differentiation (Liu et al., 2013). Therefore, this study aims to identify and characterize additional AP1 homologs in loquat to clarify their potential roles in floral induction.

The recent release of the loquat genome has provided new opportunities to explore key regulatory genes (Su et al., 2021). In this study, we identified and cloned a novel AP1-like gene, *EjAP1-like1*, from the cultivar ‘Jiefangzhong’. We investigated its sequence features, cis-regulatory elements, spatiotemporal expression patterns, responses to GA_3_ and short-day treatments, subcellular localization, and functional role via heterologous expression in *Arabidopsis*. Our results suggest that *EjAP1-like1* may function as a floral inducer in loquat. This study enriches the theoretical understanding of flowering regulation in woody perennials and provides a potential molecular target for flower-time control in loquat and other fruit crops.

## 2 Materials and methods

### 2.1 Plant materials

Buds and leaves used in this study were collected from 12-year-old fruiting trees of the loquat cultivar ‘Jiefangzhong’ grown in the *Eriobotrya* germplasm resource garden of South China Agricultural University, Guangzhou, China (23°09′N, 113°20′E) during the 2023 growing season. Samples at different developmental stages and from various tissues of the loquat plants were randomly collected at 16:00 from the upper, middle, and lower canopy positions. Apical tissues were collected at 14-day intervals from February to December. The samples included shoot apices(terminal buds containing the apical meristem) from February to September, inflorescences from September to December, and fruits from February to March. Fruits collected on March 2 and March 30 were dissected into peel, flesh, and seeds (note that the peel of the immature fruits on March 2 could not be separated). Receptacle, petal, stamen, and pistil tissues were sampled from freshly opened flowers. Leaves at different developmental stages were also collected. Stages L1–L6 represent a progression from young, tender leaves to fully expanded and mature leaves, while L7 refers to senescing yellow leaves prior to abscission. In addition, veins and mesophyll tissues were separated from L5 leaves, which were fully expanded and completely green. Tissues intended for RNA extraction were immediately frozen in liquid nitrogen and subsequently stored at –80 °C. Wild-type *Arabidopsis thaliana* (ecotype Col-0) was used for heterologous transformation, and *Nicotiana benthamiana* was used for transient expression assays. Both plant species were grown under controlled conditions of 16 h light/8 h dark photoperiod at a constant temperature of 22 °C.

### 2.2 Cloning of the *EjAP1-like1* gene

Total RNA was extracted from buds and leaves of the loquat cultivar ‘Jiefangzhong’ using the EASYspin Plus Plant RNA Rapid Extraction Kit (Aidlab), following the manufacturer’s instructions. Using 1 μg of total RNA as the template, first-strand cDNA was synthesized with the PrimeScript™ RT Reagent Kit with gDNA Eraser (Takara). The sequence of *AtAP1* (AT1G69120) was obtained from the *Arabidopsis* Information Resource (TAIR; https://www.arabidopsis.org/index.jsp). Using TBtools software (Chen et al., 2020), this sequence was aligned to the published loquat genome (Su et al., 2021), to identify the *EjAP1-like1* homologous sequence. Specific primers for cloning were designed using Primer Premier 6.0 (**Table 1**). PCR amplification was performed using PrimeSTAR^®^ Max DNA Polymerase (Takara) with the synthesized cDNA as the template. The reactions were run under the following cycling conditions: 98 °C for 2 min; followed by 30 cycles of 98 °C for 30 s, 55 °C for 30 s, and 72 °C for 1 min; with a final extension at 72 °C for 10 min and a hold at 12 °C. PCR products were confirmed by agarose gel electrophoresis, purified, and ligated into the pGEM-T Easy vector (Promega). The ligation products were transformed into Escherichia coli DH5α competent cells. Positive clones were identified by colony PCR and further verified by Sanger sequencing.

**Table 1:** Primer sequences used for cloning, qRT-PCR, and vector construction of *EjAP1-like1*.

Primer name	Sequence (5’–3’)
*EjAP1-like1*-F	ATGGGAAGAGGTAAGGTTCAGCT
*EjAP1-like1*-R	TTATATTTCATTAAAATGGCGAA
*qEjAP1-like1*-F	TGAAGCGAATCGAGAACACG
*qEjAP1-like1*-R	AAGTGCCACATCAGCATCAC
*Ejβ-actin-F*	GGATTTGCTGGTGATGATGC
*Ejβ-actin-R*	CCGTGCTCAATGGGATACTT
*EjAP1-like1*-GFP-F	GTCGACGGTATCGATAAGCTTATGGGAAGAGGTAAGGTTCA
*EjAP1-like1*- GFP-R	TCCCCCGGGCTGCAGGAATTCTATTTCATTAAAATGGCGAA

### 2.3 Gene expression analysis

qPCR primers were designed using Geneious Prime software. Quantitative real-time PCR was performed using the CFX Opus 384 Real-Time PCR System (Bio-Rad) with iTaq™ Universal SYBR Green SuperMix (Bio-Rad) as the reaction mix. Relative gene expression levels were calculated using the 2^−^ΔΔCt method. *β-actin* was used as the internal reference gene for loquat (Shan et al., 2008), and *AtPP2AA3* (AT1G13320) was used as the reference gene for *Arabidopsis thaliana* (Hong et al., 2010). Each sample was analyzed with three biological replicates and three technical replicates. Primer sequences used in this analysis are listed in **Table 1**.

### 2.4 Phylogenetic and promoter region analysis

Protein domains were predicted using the NCBI database (https://blast.ncbi.nlm.nih.gov/Blast.cgi). Multiple sequence alignment of protein sequences was performed using ClustalW in MEGA X, and a phylogenetic tree was constructed using the Neighbor-Joining (NJ) method with 1,000 bootstrap replicates. The 2,000 bp upstream sequence from the start codon of *EjAP1-like1* was retrieved from the loquat genome using TBtools software (Chen et al., 2020). Conserved cis-regulatory elements and their putative functions within the promoter region were predicted using the online PlantCARE database (accessed on 31 July 2025; http://bioinformatics.psb.ugent.be/webtools/plantcare/html/).

### 2.5 GA_3_ and short-day treatments

A GA_3_ aqueous solution was prepared at a concentration of 300 mg·L^−^¹, containing 0.1% phosphoric acid and 0.025% Triton X-100. Starting from 18 May, the solution was sprayed onto all leaves and buds every two weeks until 10 August. The control group was sprayed with distilled water. For sampling, shoot apices were collected at multiple time points, including the pre-treatment stage (27 April), the day of the first treatment (18 May), and then every 14 days thereafter.

For the short-day (SD) treatment, a shading structure was used to simulate an 8 h light/16 h dark photoperiod (light period from 10:00 to 18:00). We used a double-layer shade net for light shading, and the specific structure is shown in **Supplementary Figure S1** of Jiang et al. (2019c). Control plants were grown under natural photoperiod conditions. SD samples were collected at the same time points as the GA_3_ treatment, including the pre-treatment sampling (27 April), the day SD treatment began (11 May), and subsequently every 14 days. Three biological replicates were used for each treatment and sampling point.

### 2.6 Subcellular localization analysis

The subcellular localization of the protein was predicted using the Cell-PLoc 2.0 online tool (http://www.csbio.sjtu.edu.cn/bioinf/Cell-PLoc-2/). The *EjAP1-like1* coding sequence without the stop codon was cloned into the 35S-GFP vector to generate the fusion construct 35S-*EjAP1-like1*-GFP (primer sequences used for vector construction are listed in **Table 1**). The construct was introduced into *Agrobacterium tumefaciens* strain GV3101, and bacterial suspensions (OD_600_ ≈ 0.8) were infiltrated into the abaxial side of *Nicotiana benthamiana* leaves. After infiltration, the tobacco plants were incubated in the dark for 12 hours and then transferred to standard light conditions for 2–3 days. GFP signals were observed using a Zeiss Observer.D1 fluorescence microscope.

### 2.7 Heterologous expression of *EjAP1-like1*

The **35S:***EjAP1-like1* expression construct was introduced into *Agrobacterium tumefaciens* strain GV3101, and transformed into *Arabidopsis thaliana* ecotype Col-0 using the floral dip method (Zhang et al., 2006). Transgenic lines were selected using 0.02% Basta herbicide (10% glufosinate-ammonium solution). More than 10 independent transgenic lines were obtained, and two homozygous T_3_-generation lines were selected for analysis of flowering time and gene expression.

### 2.8 Data analysis

Statistical differences among the datasets were assessed using Student’s *t*-test. Data analysis and figure preparation were carried out with Microsoft Office 365 and GraphPad Prism 9.

## 3 Results

### 3.1 Cloning and characterization of *EjAP1-like1*

**Figure 1:**
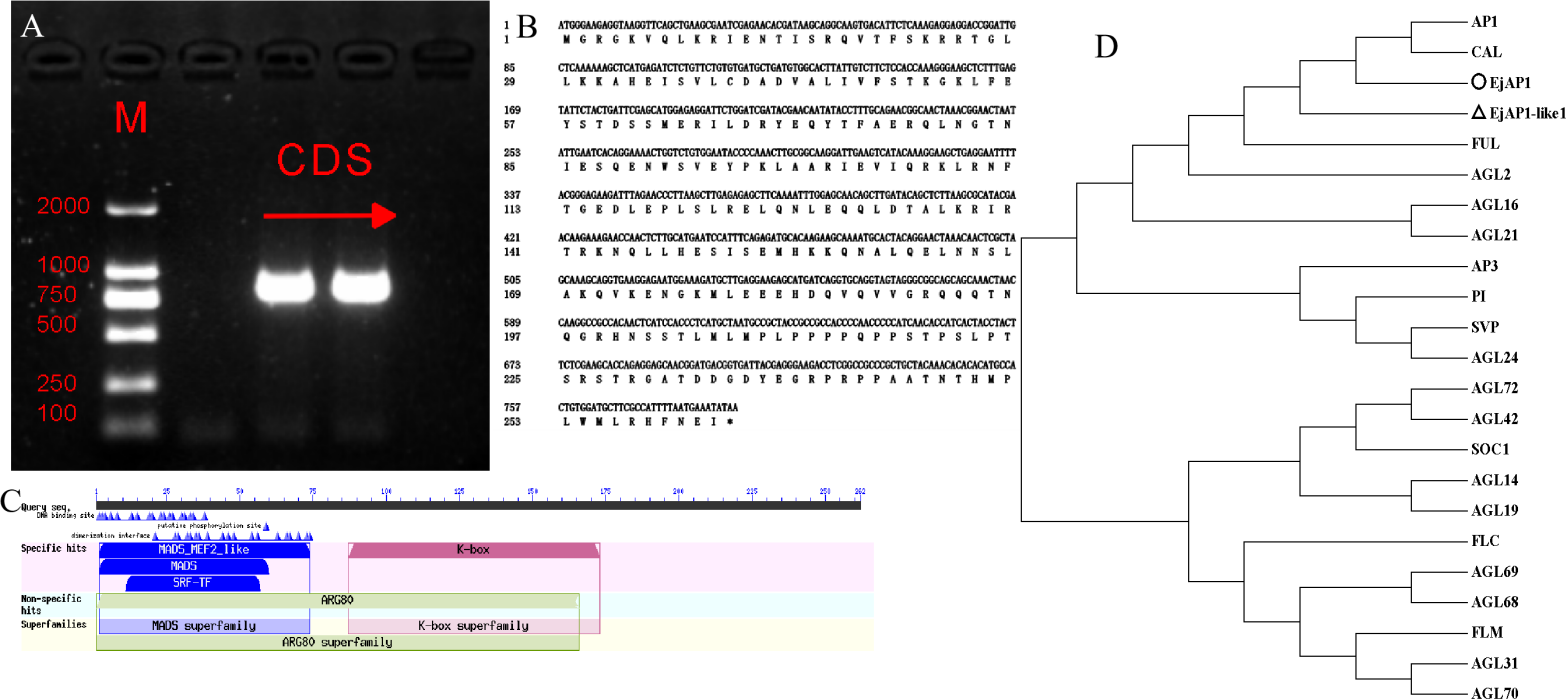
Cloning and characterization of the *EjAP1-like1* gene **(A)** Gel electrophoresis of *EjAP1-like1* PCR product,’M’ denotes the Mark DNA; **(B)** Coding sequence (CDS) of *EjAP1-like1* and its deduced amino acid sequence; **(C)** Domain structure analysis of the EjAP1-like1 protein,the blue box indicates the MADS superfamily, and the magenta box represents the K-box superfamily; **(D)** Phylogenetic analysis of EjAP1-like1, EjAP1, and members of the Type II MADS-box subfamily in *Arabidopsis thaliana* based on amino acid sequences, the arrow points to EjAP1-like1.

Using mixed cDNA from apical buds and leaves of the loquat cultivar ‘Jiefangzhong’ as a template, we amplified a fragment of approximately 750 bp corresponding to the full-length *EjAP1-like1* gene (**Figure 1A**). The PCR product was ligated into a T-vector and sequenced by a commercial service, revealing an actual length of 789 bp, which encodes a protein consisting of 262 amino acids (**Figure 1B**). To characterize the structural features of the EjAP1-like1 protein, a BLAST analysis was performed using the NCBI database. The results indicated that the protein contains conserved MADS and K-box domains, as well as a MEF2 (myocyte enhancer factor 2)-like domain, all of which are typical features of Type II MADS-box transcription factors (**Figure 1C**). Phylogenetic analysis was then conducted using members of the Type II MADS-box subfamily from *Arabidopsis thaliana*. The resulting tree showed that EjAP1-like1 is most closely related to AtAP1 (**Figure 1D**), and the gene was thus designated as EjAP1-like1. Although EjAP1-like1 shares the conserved MADS-box and K-box domains with the previously reported EjAP1 and other Rosaceae AP1 homologs, the sequence alignment and phylogenetic analyses (**Supplementary Figure S1**) revealed substantial structural divergence, indicating that EjAP1-like1 represents a distinct AP1 homolog in loquat.

### 3.2 Tissue-specific expression analysis of *EjAP1-like1*

**Figure 2:**
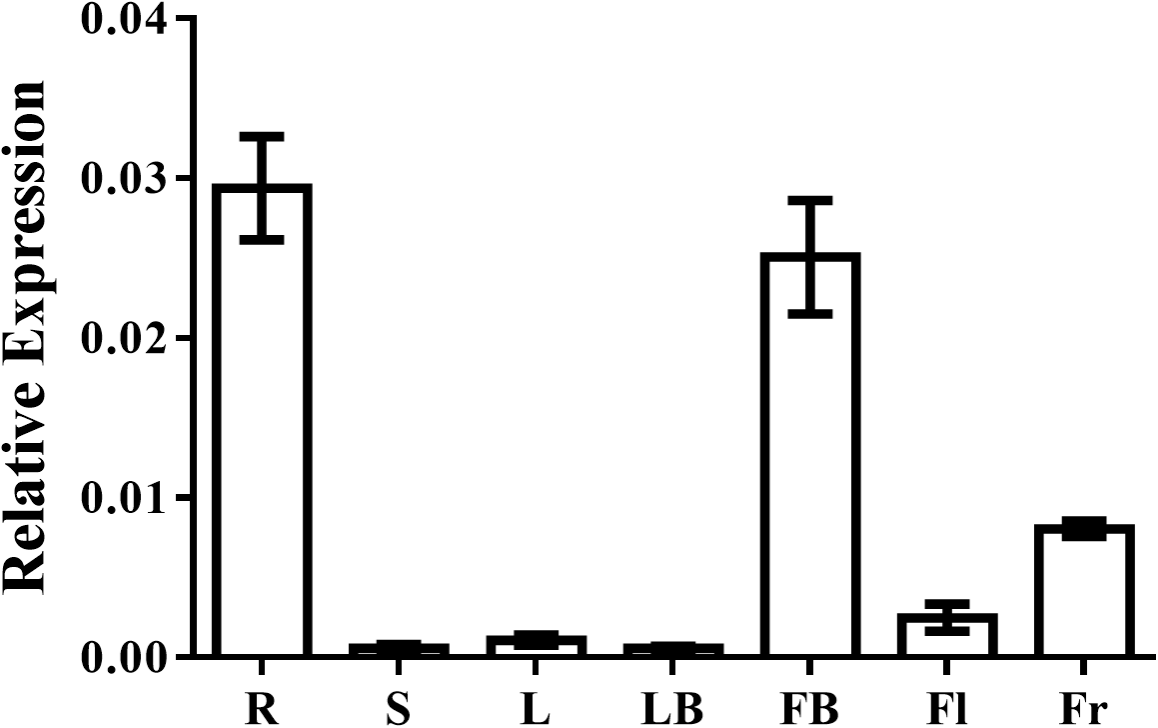
Tissue-specific expression of *EjAP1-like1* in loquat. R, root (May 26); S, stem (May 26); L, leaf (May 26); LB, leaf bud (May 26); FB, flower bud (August 18); Fl, open flower (December 8); Fr, fruit (March 30). Error bars indicating SD from three biological replicates.

To investigate the potential function of *EjAP1-like1* in loquat, we performed quantitative expression analysis across various tissues. The results showed that *EjAP1-like1* is predominantly expressed in flower buds and roots, with lower expression levels detected in fruits, open flowers, and mature leaves (**Figure 2**). Expression in other tissues was nearly undetectable. These findings suggest that *EjAP1-like1* may have a relatively conserved function in loquat, potentially similar to that of AP1 homologs in other species, and may play a role in floral bud formation.

### 3.3 Spatiotemporal expression analysis

**Figure 3:**
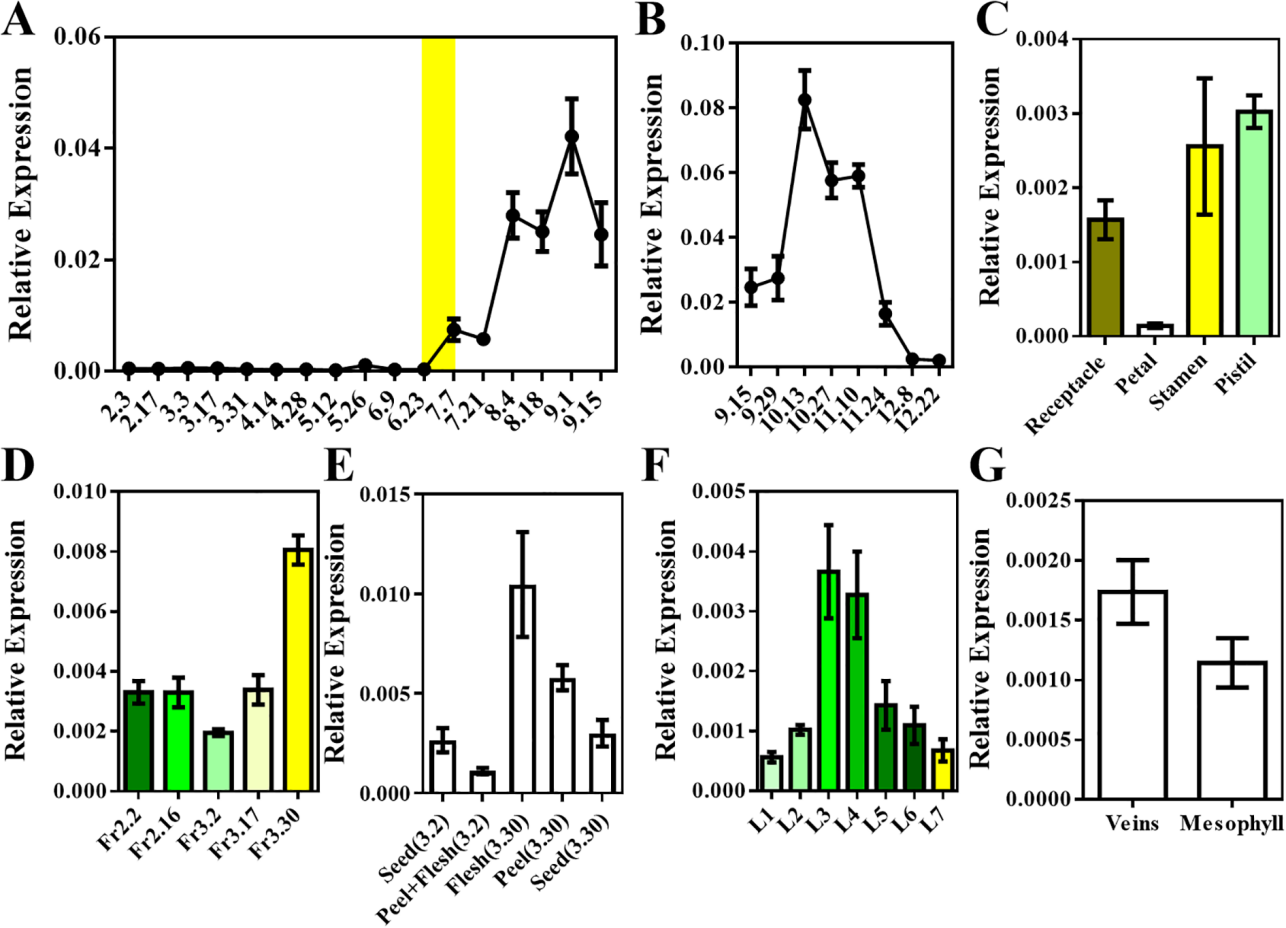
Spatiotemporal expression of *EjAP1-like1* during loquat growth and development. **(A)** Expression of *EjAP1-like1* in shoot apices, the yellow background indicates the period during which floral bud initiation occurs; **(B)** Expression pattern over floral bud development stages; **(C)** Expression in different floral organs; **(D)** Expression at various fruit developmental stages; **(E)** Expression in different fruit tissues; **(F)** Expression in leaves at different developmental stages; **(G)** Expression in different parts of the leaf. Error bars indicating SD from three biological replicates.

Previous studies have shown that floral bud differentiation in loquat begins in late June to early July. By mid-September, visible inflorescence structures can be observed at the shoot apex, flower clusters expand by mid-October, and flowering typically begins in December (Jiang et al., 2019a, 2019b, 2019c, 2025).To further explore the function of *EjAP1-like1* in loquat, we conducted a comprehensive expression analysis across different tissues and developmental stages. The results showed that in the shoot apex, *EjAP1-like1* expression began in late June to early July and exhibited a progressive increase, reaching its peak on October 13 (**Figures 3A, B**). This expression pattern is consistent with the timeline of floral bud differentiation in loquat, suggesting that *EjAP1-like1* may be involved in floral induction. In floral organs, *EjAP1-like1* was mainly expressed in the pistil, stamen, and receptacle, with the lowest expression observed in the petals (**Figure 3C**). Interestingly, *EjAP1-like1* also showed relatively high expression levels in mature fruit, particularly in the fruit flesh (**Figures 3D, E**). In leaves at different developmental stages, higher expression levels were observed during the leaf expansion phase (L3–L4), while lower levels were detected at other stages. In addition, expression was higher in leaf veins than in leaf mesophyll tissues (**Figures 3F, G**).

### 3.4 *EjAP1-like1* is repressed by GA_3_ and short-day treatments

**Figure 4:**
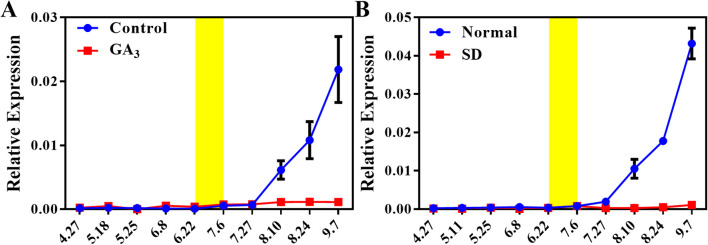
Expression of *EjAP1-like1* under different treatments. **(A)** Expression of *EjAP1-like1* in the shoot apices of GA_3_-treated and control plants; **(B)** Expression of *EjAP1-like1* in the shoot apices under short-day conditions and natural photoperiod. Error bars indicating SD from three biological replicates.

Loquat plants fail to initiate floral bud differentiation when subjected to exogenous GA_3_ application or short-day photoperiod treatment, and the detailed phenotypic responses are documented in our previous studies (Jiang et al., 2019c, 2025). Notably, our results showed that *EjAP1-like1* expression was strongly repressed under both GA_3_ and short-day conditions (**Figure 4**). However, floral bud differentiation proceeded normally in the control group. This finding further supports the notion that *EjAP1-like1* plays a key regulatory role in floral induction in loquat.

### 3.5 EjAP1-like1 is localized in the nucleus

**Figure 5:**
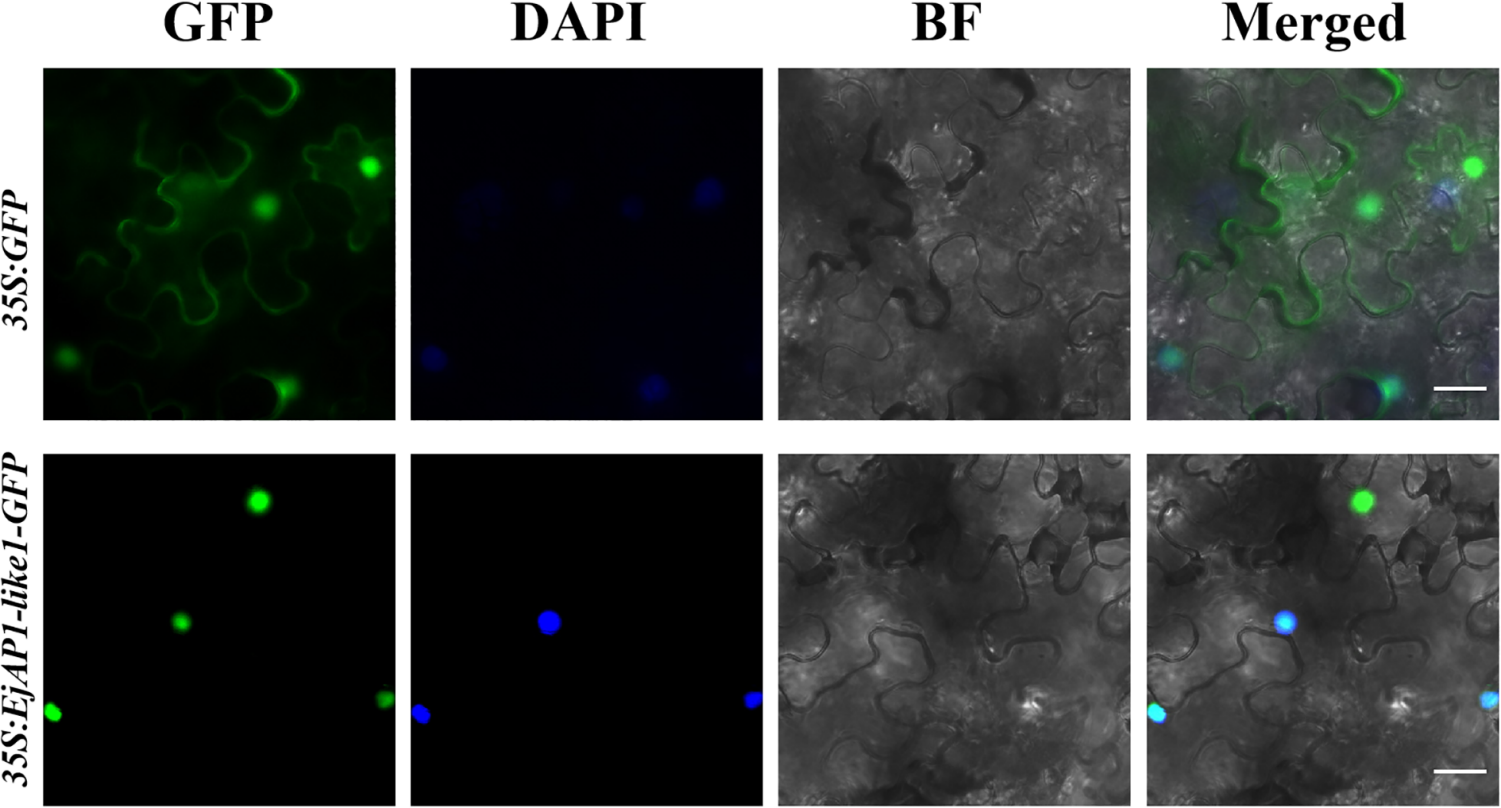
Subcellular localization of EjAP1-like1. GFP, Green fluorescent protein channel; DAPI, DAPI(4,6-diamidino-2-phenylindole, which indicates nuclear localization) channel; BF, bright-field channel; Merged, merged images of GFP, BF and DAPI stained cells; Scale bars= 20 µm.

The amino acid sequence of EjAP1-like1 was analyzed using the Cell-PLoc 2.0 online tool (http://www.csbio.sjtu.edu.cn/bioinf/Cell-PLoc-2/), and the prediction indicated that the protein is localized in the nucleus, consistent with the typical characteristics of transcription factors. To experimentally validate this prediction, we constructed a 35S:EjAP1-like1-GFP expression vector and introduced it into *Nicotiana benthamiana* leaves via *Agrobacterium*-mediated infiltration. Under fluorescence microscopy, the control vector (35S:GFP) exhibited green fluorescence signals in both the cytoplasm and nucleus, while the fusion protein (35S:EjAP1-like1-GFP) was exclusively localized in the nucleus, consistent with the in silico prediction (**Figure 5**). These results confirm that EjAP1-like1 is a nuclear-localized protein, supporting its proposed role as a transcriptional regulator.

### 3.6 Heterologous overexpression of *EjAP1-like1* promotes early flowering in *Arabidopsis*

**Figure 6:**
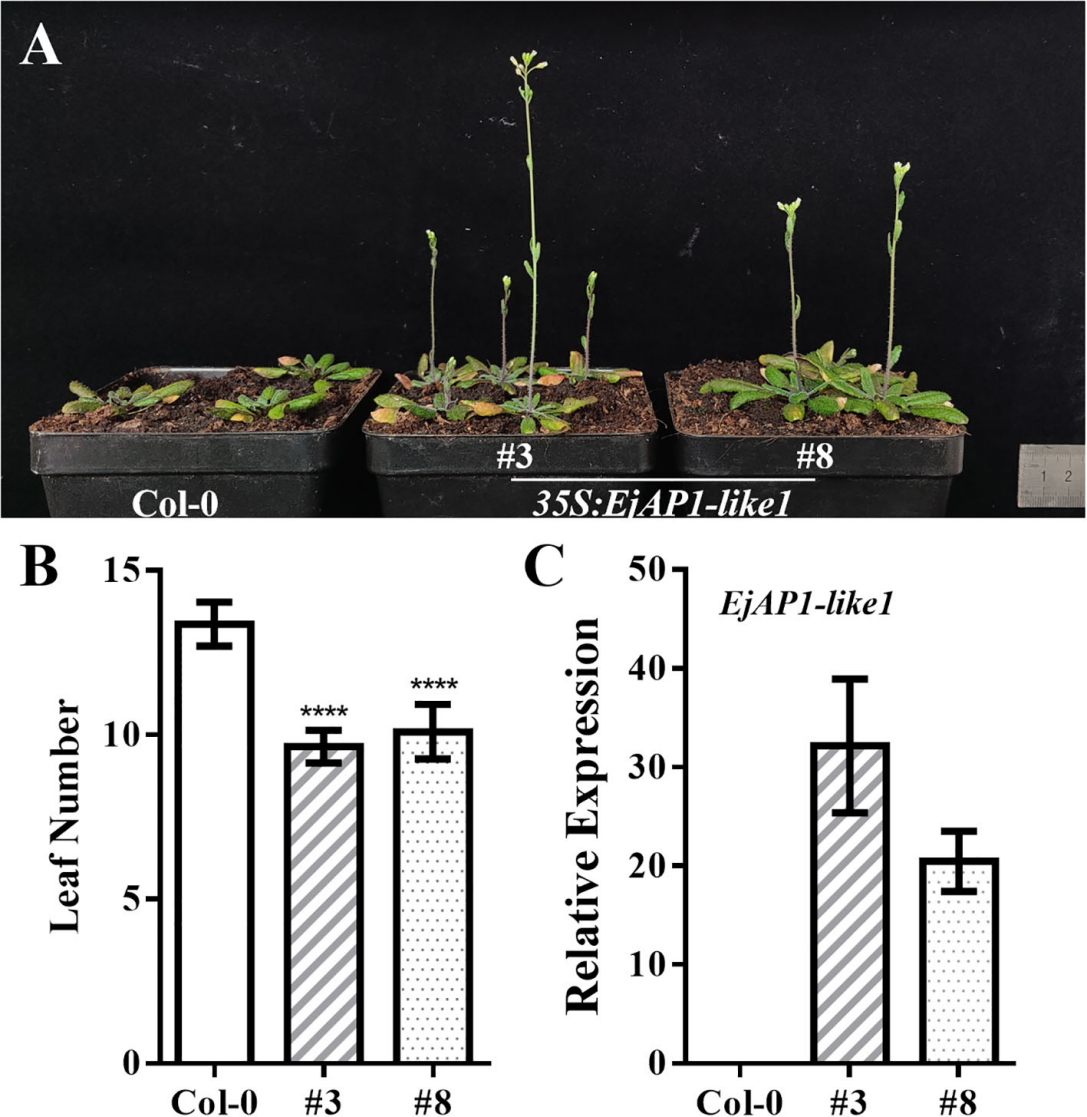
Heterologous overexpression of *EjAP1-like1* in *Arabidopsis*. **(A)** Phenotypes of wild-type Col-0 and *EjAP1-like1*-overexpressing plants; **(B)** Statistical analysis of rosette leaf number in wild-type and transgenic plants; **(C)** Expression levels of *EjAP1-like1* in Col-0 and transgenic lines. Asterisks denote significant differences between transgenic lines and Col-0 (calculated by Student’s *t*-tests), ****p < 0.0001.

To further investigate the function of *EjAP1-like1*, the gene was ectopically overexpressed in wild-type *Arabidopsis thaliana* Col-0 plants. The results showed that bolting occurred significantly earlier in transgenic lines compared to wild-type plants (**Figure 6A**). While wild-type Col-0 plants initiated bolting at the 12th, 13th, or 14th rosette leaf stage and typically bolted at approximately 25 days, the EjAP1-like1-overexpressing lines produced markedly fewer rosette leaves before bolting and flowered substantially earlier, bolting at around 18 days (**Figure 6B**).

Additionally, expression analysis confirmed that *EjAP1-like1* was not expressed in wild-type Col-0, whereas it was highly expressed in the selected transgenic lines (**Figure 6C**). These findings indicate that overexpression of *EjAP1-like1* can promote flowering in *Arabidopsis*, supporting its putative role as a positive regulator of floral induction.

### 3.7 Promoter analysis

Our previous results indicated that *EjAP1-like1* responds to GA_3_ and short-day signals and is involved in regulating plant growth and development (**Figure 4**). To further investigate the transcriptional regulation of *EjAP1-like1*, a 2,000 bp promoter region upstream of the ATG start codon was extracted from the published loquat genome (detailed sequence information provided in the Supplementary Materials). Conserved cis-acting elements in the promoter region were predicted using the PlantCARE database. The analysis revealed a total of 148 cis-acting regulatory elements within the promoter sequence. These included 30 CAAT-box elements associated with transcription initiation frequency and 34 TATA-box elements, which are core promoter motifs recognized by eukaryotic RNA polymerase II(**Table 2**).

**Table 2:** Cis-acting elements on the *EjAP1-like1* promoter.

Site name	Number	Sequence	Function
TATA-box	34	ATATAA; ATATAT; TACAAAA; TATA; TATAA; TATAAA; TATAAAT; TATAAATA; TATATA; ccTATAAAaa	core promoter element around -30 of transcription start
CAAT-box	30	CAAAT; CAAT; CCAAT; CCCAATTT	common cis-acting element in promoter and enhancer regions
Box 4	4	ATTAAT	part of a conserved DNA module involved in light responsiveness
CGTCA-motif	3	CGTCA	cis-acting regulatory element involved in the MeJA-responsiveness
TGACG-motif	3	TGACG	cis-acting regulatory element involved in the MeJA-responsiveness
P-box	3	CCTTTTG	gibberellin-responsive element
TCCC-motif	2	TCTCCCT	part of a light responsive element
G-box	2	CACGTG; GCCACGTGGA	cis-acting regulatory element involved in light responsiveness
ABRE	2	ACGTG; CACGTG	cis-acting element involved in the abscisic acid responsiveness
TCT-motif	1	TCTTAC	part of a light responsive element
ARE	1	AAACCA	cis-acting regulatory element essential for the anaerobic induction
AE-box	1	AGAAACAA	part of a module for light response
CCAAT-box	1	CAACGG	MYBHv1 binding site
A-box	1	CCGTCC	cis-acting regulatory element
MBS	1	CAACTG	MYB binding site involved in drought-inducibility
GT1-motif	1	GGTTAA	light responsive element
G-Box	1	CACGTG	cis-acting regulatory element involved in light responsiveness
MRE	1	AACCTAA	MYB binding site involved in light responsiveness
Other	64	–	Function not described

Beyond these fundamental motifs, we further focused our analysis on functionally relevant cis-elements associated with flowering, hormone signaling, and stress responses. Specifically, the promoter contains six MeJA-responsive motifs (three TGACG-motifs and three CGTCA-motifs), thirteen light-responsive elements (including Box 4, TCCC-motif, G-box, TCT-motif, MRE, and GT1-motif), three GA-responsive P-box elements, and one MYB binding site implicated in drought induction (**Table 2**). These key regulatory elements suggest that *EjAP1-like1* transcription may be modulated by multiple environmental and hormonal cues, including light, gibberellin, MeJA, and drought stress.

## 4 Discussion

Loquat (*Eriobotrya japonica*) is a unique fruit tree species that flowers in autumn and winter and matures in spring, providing a distinct market advantage due to its off-season fruit supply. However, low temperatures and the risk of frost during winter pose significant threats to both yield and fruit quality (Peng et al., 2022). Therefore, elucidating the regulatory mechanisms underlying floral induction and identifying key genes involved are crucial for enabling precise control of flowering time, ensuring yield stability, and advancing molecular breeding strategies. In this study, we identified and cloned a novel AP1 homolog in loquat, designated *EjAP1-like1*. Through a combination of molecular and functional analyses, we provide preliminary evidence that *EjAP1-like1* acts as a positive regulator during floral bud differentiation. Moreover, its expression pattern suggests that it may be responsive to environmental signals, highlighting its potential role in mediating developmental responses to external cues.

As a key member of the MADS-box transcription factor family, the AP1 gene plays a central role in floral organ development and the regulation of flowering time (Alejandra Mandel et al., 1992; Mandel and Yanofsky, 1995; Becker and Theißen, 2003). Homologs of AP1 have been isolated from a wide range of plant species, including pea (*Pisum sativum*), apple (*Malus domestica*), wheat (*Triticum aestivum*), *Phalaenopsis* orchid (‘Hatsuyuki’), longan (*Dimocarpus longan*), trifoliate orange (*Poncirus trifoliata* L. Raf.), birch (*Betula platyphylla × Betula pendula*), and poplar (*Populus tomentosa*). In these species, AP1-like genes consistently exhibit functions related to floral promotion or floral meristem identity regulation (Berbel et al., 2001; Kotoda et al., 2002; Adam et al., 2007; Song et al., 2011; Winterhagen et al., 2013; Huang et al., 2014; Sun et al., 2014; Chen et al., 2015; Ma et al., 2025; Yan et al., 2025). Our findings are consistent with previous studies in several key aspects: the conserved domain architecture of *EjAP1-like1*, its floral-promoting effect when ectopically expressed in *Arabidopsis*, and its high expression levels in floral buds and floral organs all align with the established functions of AP1-like genes in other species. Notably, *EjAP1-like1* also exhibits appreciable expression in mature fruits and leaves at specific developmental stages, suggesting that it may additionally participate in fruit development or in the regulatory crosstalk between vegetative and reproductive organs. This observation is in line with studies in apple and tomato, where AP1-like genes have also been implicated in fruit developmental processes (Vrebalov et al., 2002; Zhang et al., 2024; Yan et al., 2025). Another interesting finding of this study is the relatively high expression of *EjAP1-like1* in root tissues. This observation suggests that *EjAP1-like1* may have acquired species-specific or neofunctionalized roles beyond its canonical function in floral meristem identity in loquat, a possibility that warrants further investigation.

Gibberellins (GAs) regulate floral development by modulating the expression of floral meristem identity genes such as AP1 and LFY through the action of DELLA proteins (Yu et al., 2004). In the light signaling pathway, CONSTANS (CO) mediates the photoperiod-dependent regulation of FT expression, and the FT–FD complex subsequently induces AP1 expression at the shoot apex, thereby initiating floral development (Abe et al., 2005). In this study, the promoter analysis of *EjAP1-like1* revealed the presence of multiple cis-acting elements responsive to environmental cues, including light, gibberellin (GA), methyl jasmonate (MeJA), and drought. This suggests that *EjAP1-like1* may function as an integrator of environmental signals to modulate flowering time. While these results provide a useful framework, it should be noted that the analysis is predictive and descriptive in nature, as it was based on in silico motif identification using PlantCARE. Functional validation—such as electrophoretic mobility shift assays (EMSAs), yeast one-hybrid assays, or promoter–reporter studies—will be essential in future work to establish the biological significance of these motifs. Our experimental results further confirmed that *EjAP1-like1* responds to exogenous GA_3_ and short-day treatments. However, the underlying mechanisms by which GA_3_ and short-day affect *EjAP1-like1* remain unclear, as current findings are limited to expression-level changes without deeper exploration into the upstream regulatory pathways.

Regarding its expression profile, *EjAP1-like1* exhibited sustained high expression during the critical floral bud differentiation stage in loquat (from late June to October), with localization predominantly in the shoot apex and floral organs. This expression pattern is similar to that reported previously for other loquat AP1 homologs, including *EjAP1, EjAP1-1*, and *EjAP1-2* (Liu et al., 2013; Jiang et al., 2025), and aligns well with the unique phenological characteristics of loquat, which undergoes floral induction in autumn and flowering in winter. Although *EjAP1-like1* showed low relative transcript levels in our qRT-PCR assays, relative expression values cannot be used to infer the magnitude of gene function; nevertheless, its stage-dependent expression trend provides biologically meaningful clues to its potential role during floral meristem and organ identity development. Notably, Jiang et al. (2025) reported that floral initiation in loquat is regulated through the classical FT–FD–AP1 module, raising the possibility that EjAP1-like1 may function within a similar regulatory framework. However, whether *EjAP1-like1* participates directly in FT-mediated pathways, or acts in parallel with other key flowering regulators such as *EjSOC1* and *EjFT*, remains to be clarified. These findings suggest that *EjAP1-like1* may serve as an early molecular marker for floral initiation, although the upstream signaling events—particularly GA_3_- and SD-mediated regulation—were assessed only at the transcriptional level in this study. Future work integrating biochemical and genetic approaches will be required to elucidate its precise regulatory relationships and upstream control mechanisms.

## 5 Conclusions

In summary, this study is the first to characterize the molecular features and biological function of *EjAP1-like1* in loquat. Our findings demonstrate that *EjAP1-like1* plays a crucial role in promoting floral initiation and is regulated by both endogenous cues and environmental signals. Future studies involving transgenic manipulation, promoter functional assays, and interaction analyses with key flowering regulators such as *FT* and *SOC1* will further clarify the molecular network through which *EjAP1-like1* controls floral development. Importantly, the early activation and floral-inducing capability of *EjAP1-like1* highlight its potential as a molecular target for regulating flowering time and developing breeding strategies in loquat. These insights not only deepen our understanding of flowering regulation in this evergreen fruit tree but also provide a theoretical foundation for applying similar approaches to flowering time manipulation in other woody perennial species.

## Data Availability

The raw data supporting the conclusions of this article will be made available by the authors, without undue reservation.
