# Supplementary material for: Functional characterization of EjAP1-like1 reveals its role in floral development and flowering time regulation in loquat (Eriobotrya japonica)
Source: Front Plant Sci. 2025 Dec 12;16:1713266. doi: 10.3389/fpls.2025.1713266 (PMC12741147; doi:10.3389/fpls.2025.1713266)
Supplement: Supplementary file 1 [file DataSheet1.docx]

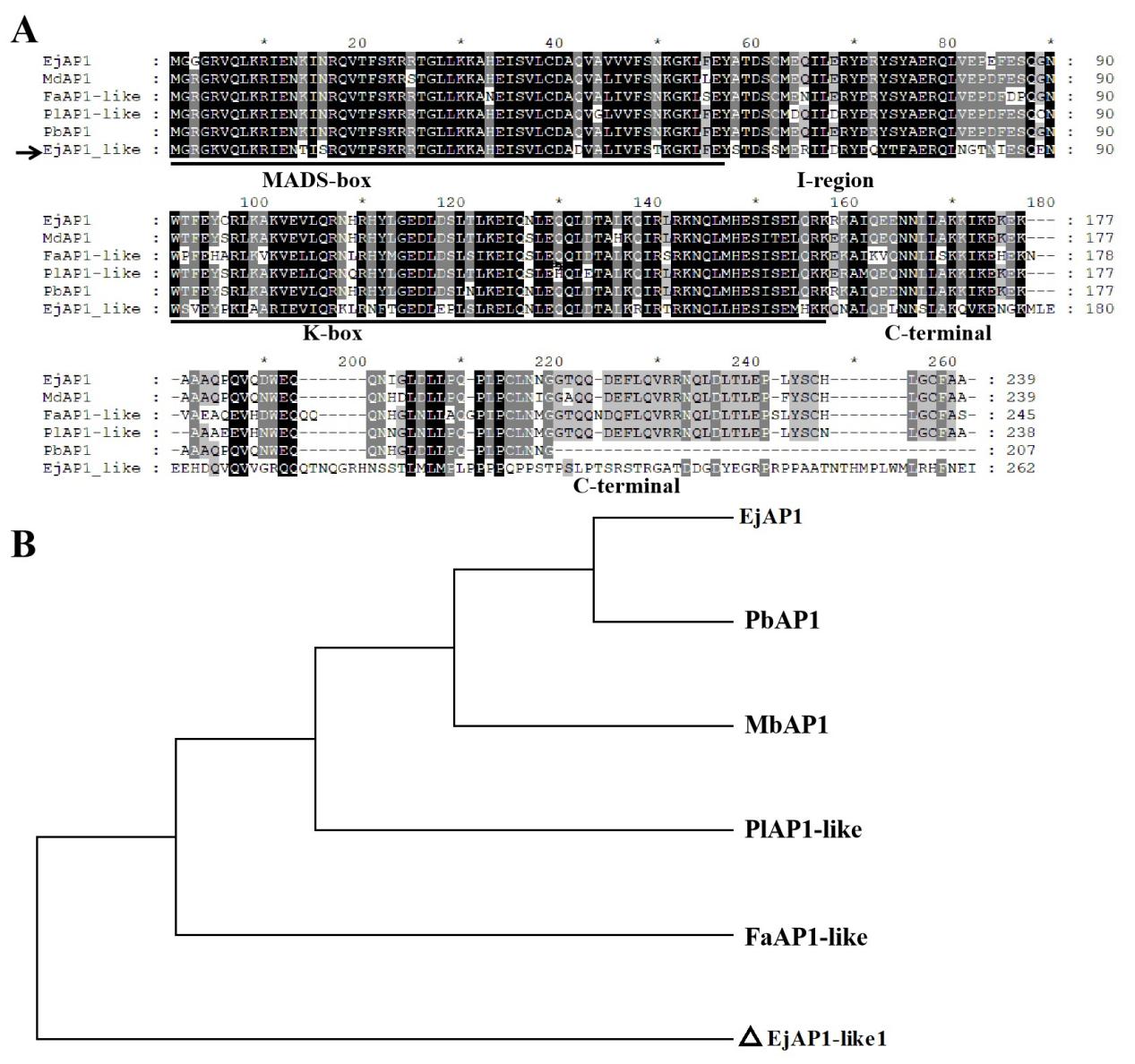


**FIGURE S1** Amino acid sequence alignment and phylogenetic analysis of EjAP1-1 and AP1 homologs from other Rosaceae species. (A) Multiple sequence alignment of AP1 proteins; (B) Phylogenetic analysis of AP1 homologs. The protein sequences of AP1 homologs used in this study were retrieved from NCBI. Accession IDs: EjAP1(AAX14151.1); MdAP1(ACD69426.1); PbAP1(XP_048441156.1) ; FaAP1-like(AFA42327.1); PlAP1-like(ACT67688.1).

>EjAP1-like1

ATGGGAAGAGGTAAGGTTCAGCTGAAGCGAATCGAGAACACGATAAGCAGGCAAGTGACATTCTCAAAGAGGAGGACCGGATTGCTCAAAAAAGCTCATGAGATCTCTGTTCTGTGTGATGCTGATGTGGCACTTATTGTCTTCTCCACCAAAGGGAAGCTCTTTGAGTATTCTACTGATTCGAGCATGGAGAGGATTCTGGATCGATACGAACAATATACCTTTGCAGAACGGCAACTAAACGGAACTAATATTGAATCACAGGAAAACTGGTCTGTGGAATACCCCAAACTTGCGGCAAGGATTGAAGTCATACAAAGGAAGCTGAGGAATTTTACGGGAGAAGATTTAGAACCCTTAAGCTTGAGAGAGCTTCAAAATTTGGAGCAACAGCTTGATACAGCTCTTAAGCGCATACGAACAAGAAAGAACCAACTCTTGCATGAATCCATTTCAGAGATGCACAAGAAGCAAAATGCACTACAGGAACTAAACAACTCGCTAGCAAAGCAGGTGAAGGAGAATGGAAAGATGCTTGAGGAAGAGCATGATCAGGTGCAGGTAGTAGGGCGGCAGCAGCAAACTAACCAAGGCCGCCACAACTCATCCACCCTCATGCTAATGCCGCTACCGCCGCCACCCCAACCCCCATCAACACCATCACTACCTACTTCTCGAAGCACCAGAGGAGCAACGGATGACGGTGATTACGAGGGAAGACCTCGGCCGCCCGCTGCTACAAACACACACATGCCACTGTGGATGCTTCGCCATTTTAATGAAATATAA

>aa

MGRGKVQLKRIENTISRQVTFSKRRTGLLKKAHEISVLCDADVALIVFSTKGKLFEYSTDSSMERILDRYEQYTFAERQLNGTNIESQENWSVEYPKLAARIEVIQRKLRNFTGEDLEPLSLRELQNLEQQLDTALKRIRTRKNQLLHESISEMHKKQNALQELNNSLAKQVKENGKMLEEEHDQVQVVGRQQQTNQGRHNSSTLMLMPLPPPPQPPSTPSLPTSRSTRGATDDGDYEGRPRPPAATNTHMPLWMLRHFNEI*

>EjAP1-like1-pro

TCACATCCCGGCCCGGGCGCACCACCACAACCCAGGCCCGACTCCACCGTAGCACGATATTGTCCGCTTTGGGCCCCAACCACGCCCTCACGGTTTTGTTTCTGGGAACTCACACGAGAACTTCCCAGTGGGTCACCCATCATGGGAATGCTCTCGCGCGCTACTCGCTTAACTTCGGAGTTCCGATGGAACCCGAAGCCAGTGAGCTCCCAAAAGGCCTCGTGCTAGGTAGAGATGAGAATATACATATAAGGATCACTCCCCTGGGCGATGTGGGATCTTACAATCCACCCCCCTTAGGGGCCCAACTTCCTCGTCGGCACACACGCGGCCAGGGTTAGGCTCTGATACCAAATTGTCACATCCCGGCCCGGGCGCACCACCACAACCCAGGCCCGACTCCACTGTAGCACGATATTGTCCGCTTTGGGCCCCAACCACGCCCTCACGGTTTTGTTCTGGGTTCTCACCCACTCACTGCGCATTCTGATGCTTAGGCAGCTTTAGGTTTAAATCTATTCATATTTTTCCACACCTTATGGCTTCGTCACCGTCCAGGTGTCGGCCAGCACATCTCGATTCGATGTCCACGTGGATATTTCAGGTCAGGGTGTGTCAGATCGACACCGAATTAAGTTGATATAAAGTTTTCCATTAATCAATATAACACTAAAAACACGCATATTGAGAGTGTATGTAACAAAAGGATAATAATTAAAACATATTATTCGGTTATTCTACAACACAACTATTTTTTTTTAAAGTACAAACAATTTTTTTTTGTTCATTTAGTTTTAGTGGTGATTAAAAAAAAGCTCCAAATTAAACTGATCAATTTGGGTTTGGTGCTTTCATTCATTTTTTTTTTTTTAGAACAAAGGCCTATATAGTACTCCTAAAGAAAAATGGTTGCATGTGCCAAGTGCCACCAAATGAAAGGGCGTCCTAGACATATAGAGGAGTTATCGAATTCTCGCTTTTTGTTAACAAAATGAGTTGAAATCCCCTTAAAAAGTGGTCACATGCTGTTTCATTAATTTCTTCTATTGAATGTGACTTTTTGTTAATAAATACACTTTCACACATAAAGGACAAACTTGGAGAGGAAATTAAGTTGAAGGGCGAAAGTGGAAAAGCACGAAGTGTTTAGGAGGGCAATACGAAATTAACATTCTACCAGTATATTTCAGGGAGATTTAACAAAACACTTCCGGTACTGTTTACTTTTAATGAAAAACCATATTTATATGTTTTTCTGGTACTATTCACCATACCTTTAAAAAGGACTTTTCATTAAAAATGAAGTTTTTTTTGGACTTTTCGTTAGTGTTCCTTATATTTCATCAAGAGATAGGCCATATAAATTTGGATGGTTAGAAATATAAAGTTATAATTCATACTTAATTAATCTTTGGTTGGAATAATTAATAAAGCTTAGAAGTGACGTCAGCAACGGCAAATGCATACCATGCTCCTATAATGCCTTCCGTTTGGTCCCGCAGAGCTTAACCTGACAAGATATTTGTACGGACTGTCTGCAATCCTGTGACTAAACTGAGACACTAAGCAACTGAATTGGCCTTTTGGACCACTATCAATTCGACAACGCATTACCCCAATACCACCAGGTTTCCCAAAACTACCATTCTCTCTCTCTCTTTCTCTCTCTCTCTCTCTCTCTCTCCCCATATTTATGCACAGCGCCTTCGCTTCCACAAAACCTCCCAATACAAAAATAGAAAGACACTACAAAAAAAGCCCCTACAGAAAACGCCCCTTCTTTCTTCCTTATCATGCTTTTGCTTTTTATCATCATCCAGCTGCTTCCTTTCCTTTCTTGTATGAACCAAATTTAGGGTTTTCTCTCAAATTGGGTATATTTTCAATCTCTTGGGTTTCTAAGCACTAAAAAAGCAAGTGGTACTTCTTCTTCTCCCTCCTTTCTATATTTAGCAATATATAAGGGGGAAAAAAAGAAAGGGAAAATAAAAAGGAAAAAAATG
